# Anomalous Origin of Left Coronary Artery from the Pulmonary Trunk in a Mildly Symptomatic Adult Female

**DOI:** 10.1155/2013/840741

**Published:** 2013-10-10

**Authors:** Kevin Fan-Ying Tseng

**Affiliations:** Department of Surgery, Ten Chen General Hospital, Chungli, Taoyuan County, Taiwan

## Abstract

Anomalous origin of the left coronary artery from the pulmonary trunk, also known as Garland-Bland-White syndrome, is an extremely rare but potentially fatal congenital cardiovascular anomaly, and it often exists as an isolated condition. We hereby report an adult female who was admitted for mild chest discomfort and was accidentally diagnosed to have anomalous origin of the left coronary artery from the pulmonary trunk. This anomaly was simply repaired by using a bovine pericardial patch to obliterate the anomalous opening in the pulmonary trunk and a single coronary artery bypass graft. This report highlights the characteristic events of the anomaly in an adult with only mild symptoms.

## 1. Introduction

Anomalous origin of the left coronary artery from the pulmonary trunk, also known as Garland-Bland-White syndrome, is an extremely rare but potentially fatal congenital cardiovascular anomaly, and it often exists as an isolated condition. Some reports have found its association with tetralogy of Fallot (TOF), complete atrioventricular septal defect, and aortopulmonary (AP) window, which arises secondary to failure of septation in the aorticopulmonary trunk [1–10].

## 2. Case Report

The patient is a 36-year-old female school teacher who was in her normal state of health until two years prior to the present admission when the patient started to feel occasional pressure-like chest discomfort and palpitation. The patient went to a local hospital for help. Mild mitral regurgitation was told, and the patient was then discharged with medical management as an outpatient. However, one month prior to the present admission, the patient's chest tightness, although still mild on onset, had increased in frequency. The patient went back to the same hospital for further evaluation. This time, a cardiac catheterization was performed and accidentally revealed anomalous drainage of left coronary artery (LCA), including all its branches (like the left anterior descending artery, left circumflex artery, and the left diagonal artery), into the main pulmonary artery. Single right coronary artery (RCA) originated from the aorta, and mild mitral regurgitation was seen concurrently in the cardiac catheterization. Upon recommendations from the patient's family, the patient came to our hospital and was admitted for surgical managements.

**Figure 1:**
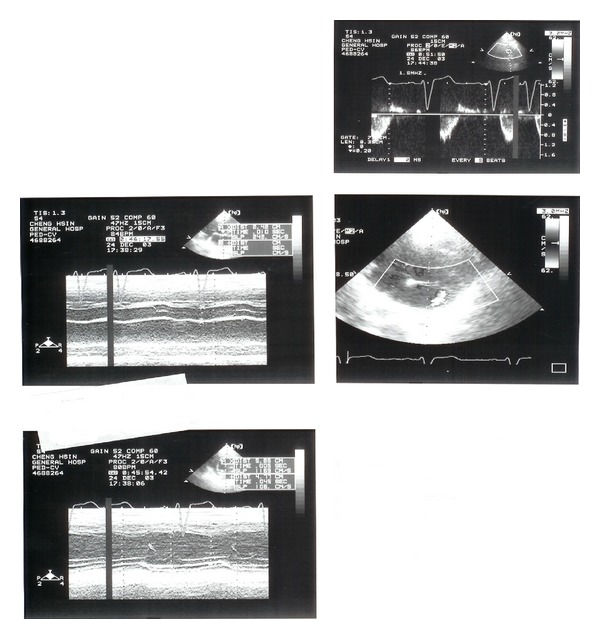
Echocardiographic representation indicating the abnormal opening of the left coronary artery inside the main pulmonary trunk.

After the patient was admitted into our service, chest X-ray, ECG, carotid ultrasound, pulmonary function test, pressure volume recording, blood analysis, and viral check were done and all revealed normal findings. No fever, leukocytosis, or any sign that might reveal infectious process had been determined. The patient was clear for surgical correction (Figure 1).

**Figure 2:**
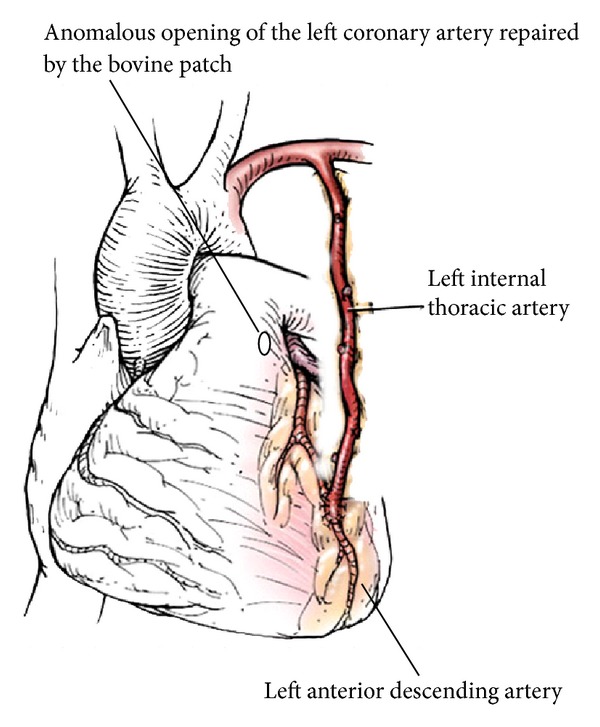
Illustrated representation of the corrective procedure for the anomalous left coronary artery. A bovine pericardium patch was applied into pulmonary trunk to obliterate the anomalous opening of the left coronary artery, while the left internal thoracic artery was used for the anastomosis to the left anterior descending artery.

The patient received surgery on December 25, 2003 (Christmas Day). The operation consisted of closing the anomalous opening in the pulmonary trunk simply with a commercially available bovine pericardium patch; concurrently, a single coronary artery bypass utilizing the Left Internal Thoracic Artery was made to the patient's Left Interventricular Artery (left anterior descending artery). The patient tolerated the procedure well. The anastomosed artery was first perfused from both the antegrade and the retrograde aspects. Vessel flow meter was then used to check the blood flow of the anastomosed artery. Good and adequate flows (mean flow: 93.1 mL/min with no insufficiency) revealed on the flow meter data. The patient's postoperative recovery phase was smooth with no complication occurred. The patient was transferred to the general ward after just four days in the intensive care unit, and she was discharged ten days after the operation (Figure 2).

## 3. Discussion

Anomalous origin of the left coronary artery from the pulmonary artery (ALCAPA) was first described by Brook in 1886 [11], and, in 1933, Bland, White, and Garland reported the clinical syndrome of this disease for the first time [1–3, 7, 10, 12–15]. Its occurrence is rare (present in one of 300,000 live births or about 0.26% in patients with congenital heart disease) [1, 2, 7, 10, 16]. Ever since its first introduction, it was generally an isolated genetic anomaly although there are reports of its association with other diseases including ventricular septal defect, patent ductus arteriosus, tetralogy of Fallot, and AP window [2, 5, 6, 10]. Anatomically, the whole left main coronary artery or only the left anterior descending or circumflex branch connects anomalously to the proximal pulmonary trunk or very rarely to the proximal right pulmonary artery. Very rarely, both coronary arteries connect to the pulmonary artery by a single trunk [3, 7, 13, 15]. The anomalous main LCA connects most often to the sinus of valsava immediately above the left or posterior cusp of the pulmonary trunk and rarely above the right cusp [2, 7]. Branching pattern of the anomalous left coronary artery remains normal. Because of its high mortality rate (up to 90%) [2], early diagnosis and prompt surgical interventions are necessary to provide gradual myocardial recovery and good clinical outcome, and, due to this reason, the majority of the cases were diagnosed before the patient reaches the age of one year. But even with clinical awareness and early intervention, 65% of the infants born with this anomaly die within the first year of life [7, 10, 16]. In the remaining infants who survived beyond the age of one year, the hazard lessens considerably, and the chronic phase ensued. Among the patients who are in the chronic phase and lived into the adulthood, as in the patient reported in this case, rich collateral from the right coronary artery, which arises normally from the aorta, feeds the left coronary artery and the flow is reversed, in which the left coronary artery drains into the pulmonary artery [1, 2, 7, 10, 12, 17, 18]. Many such patients are in good health, and few even have normal ECGs. Survival beyond the first year may be related to marked RCA dominance, supplying not only the diaphragmatic portion of LV but also much of the septum and lateral wall [7]. Patient with these arrangements may occasionally only have papillary muscle ischemia and fibrosis, and mitral regurgitation may dominate the clinical picture [7, 13]. As with the patient reported in this case, the echocardiography revealed mitral regurgitation, and the cardiac catherization showed RCA dominance. Collateral circulation from the RCA is adequate to prevent infarction since few patients presenting later in life give a history of hospitalization [1, 2, 6, 16–18]. Some adults remain asymptomatic or complain only of fatigue, dyspnea, or palpitations. About half have effort angina. The resting ECG is always abnormal, with ST changes or evidence of old anterolateral infarction [3, 7]. Exercise ECG usually shows an abnormal ischemic response, while the stress thallium myocardial imaging is usually abnormal [7]. CXR may be normal or may show cardiac enlargement. Cineangiography shows collaterals from the RCA and usually a near normal LV ejection fraction, but with anterolateral hypokinesia [7, 8, 10, 16]. However, patients who survived infancy continue to be at risk of death from chronic heart failure secondary to ischemic left ventricular cardiomyopathy, and diagnosis is an indication for operation even among the older patients [7]. In older patients, internal thoracic artery grafting with or without mitral valve repair is a reasonable alternative when size of the graft permits, and this case is achieved simply by the standard coronary arterial bypass grafting technique and ligation or obliteration of the anomalous left coronary artery [2, 7–9, 15, 18–22]. The risk or premature death depends on the perioperative status of the left ventricle (especially the left ventricular myocardium) and mitral regurgitation [2, 7, 13–16, 19, 21, 22]. The postoperative functional class depends primarily on preoperative LV status and it was generally good late postoperatively. The LV, size (including cardiothoracic ratio) is nearly always markedly reduced after operation [7]. Signs of myocardial ischemia are also reduced. However, the myocardial flow reserve is reduced, and exercise tolerance is lower than normal among the survivors [2, 7, 10, 13–16, 19, 21, 22]. In terms of mitral regurgitation, when operation is performed in infancy, even important mitral regurgitation can regress postoperatively. However, if the mitral regurgitation was severe before surgery, it would not regress, and reoperation would require a few months to a few years later [2, 7, 14, 16, 22]. Fortunately, in our patient, the preoperative echocardiography indicates only mild mitral regurgitation, and its severity was reduced after the operation, which rendered the patient into a low reoperation risk. In addition, the patient's left ventricular status was not severely impaired prior to the operation, and this might be the main contributing cause for the patient's fast and uneventful recovery. 

## 4. Conclusion

Anomalous origin of the left coronary artery from the pulmonary trunk, also known as Garland-Bland-White syndrome, is an extremely rare but potentially fatal congenital cardiovascular anomaly. With its high mortality within the first year of life, even fewer infants who were born with this anomaly. Our case was thereby presented not only because of its rarity but also for the mild clinical onset the patient presented, and a simple operation utilizing the well-established and common cardiac procedures can effectively correct this highly fatal defect.
